# Review: elimination of bacteriophages in whey and whey products

**DOI:** 10.3389/fmicb.2013.00191

**Published:** 2013-07-16

**Authors:** Zeynep Atamer, Meike Samtlebe, Horst Neve, Knut J. Heller, Joerg Hinrichs

**Affiliations:** ^1^Department of Dairy Science and Technology, Institute of Food Science and Biotechnology (150e), University of HohenheimStuttgart, Germany; ^2^Department of Microbiology and Biotechnology, Max Rubner-Institut, Federal Research Institute of Nutrition and FoodKiel, Germany

**Keywords:** bacteriophages, dairy technology, whey recycling, inactivation, resistance

## Abstract

As the cheese market faces strong international competition, the optimization of production processes becomes more important for the economic success of dairy companies. In dairy productions, whey from former cheese batches is frequently re-used to increase the yield, to improve the texture and to increase the nutrient value of the final product. Recycling of whey cream and particulated whey proteins is also routinely performed. Most bacteriophages, however, survive pasteurization and may re-enter the cheese manufacturing process. There is a risk that phages multiply to high numbers during the production. Contamination of whey samples with bacteriophages may cause problems in cheese factories because whey separation often leads to aerosol-borne phages and thus contamination of the factory environment. Furthermore, whey cream or whey proteins used for recycling into cheese matrices may contain thermo-resistant phages. Drained cheese whey can be contaminated with phages as high as 10^9^ phages mL^-1^. When whey batches are concentrated, phage titers can increase significantly by a factor of 10 hindering a complete elimination of phages. To eliminate the risk of fermentation failure during recycling of whey, whey treatments assuring an efficient reduction of phages are indispensable. This review focuses on inactivation of phages in whey by thermal treatment, ultraviolet (UV) light irradiation, and membrane filtration. Inactivation by heat is the most common procedure. However, application of heat for inactivation of thermo-resistant phages in whey is restricted due to negative effects on the functional properties of native whey proteins. Therefore an alternative strategy applying combined treatments should be favored – rather than heating the dairy product at extreme temperature/time combinations. By using membrane filtration or UV treatment in combination with thermal treatment, phage numbers in whey can be reduced sufficiently to prevent subsequent phage accumulations.

## INTRODUCTION

Various strategies have been implemented in dairies to minimize the risk of fermentation failures caused by bacteriophages in the dairy industry (Guglielmotti et al., 2012; Marco et al., 2012). Heat treatment processes at defined temperature/time combinations have been used to inactivate intrinsic thermo-resistant phages in milk, whey, or whey products (Atamer et al., 2009, 2011; Atamer and Hinrichs, 2010). In modern cheese making, recycling of whey components (i.e., whey proteins in particulated form and whey cream) and their incorporation into cheese milk is frequently done to improve its nutrient value as well as the economic effectiveness of cheese production (Lawrence, 1993; Hinrichs, 2001). However, the re-use of native whey preparations as an ingredient of fermented milk products still implies the peril of phage contamination of dairy environments. Therefore, phage elimination procedures are pivotal in cases of recycling of whey and utilization of whey powders in fermented products such as yogurt and fresh cheese (Penna et al., 1997; Tamime and Robinson, 1999). Fermentation disturbances can be unpredictable, making the production process unstable. In general, a total failure of fermentation batches does not occur, when mixed-strain starter cultures and culture rotation regimes are used. Yet, delays in production and variations in product quality are frequently encountered. Moreover, entire fermentation vats with large volumes of 20,000 to 50,000 L or – in particular – batches produced on the subsequent days may be harmed severely (Kleppen et al., 2011), when whey supplements contaminated by phages are used. For the elimination of phages in whey and whey products, both the reliable inactivation of heat-resistant phages and furthermore the preservation of native whey proteins are crucial challenges.

## THERMAL STABILITY OF DAIRY PHAGES

**FIGURE 1:**
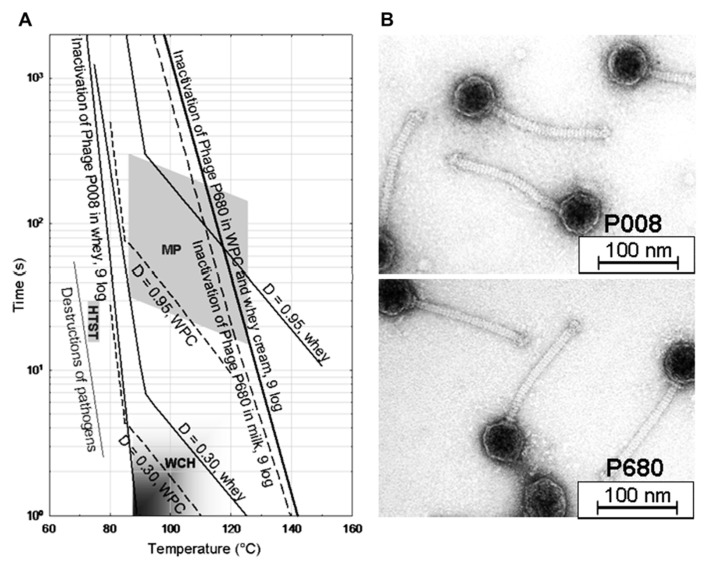
** Temperature–time graph of heat treatment of whey protein concentrate (5.3% protein) and skim milk required for 9-log inactivation of *Lactococcus lactis* phages P680 (heat-resistant) and P008 (heat-sensitive) (A)**. MP, microparticulation process of whey proteins; WCH, whey cream heating; HTST, high temperature/short time pasteurization; D, degree of β-lactoglobulin B denaturation; WPC, whey protein concentrate (Atamer and Hinrichs, 2010). Transmission electron micrographs of both phages are also shown **(B)**.

*Lactococcus lactis* phages have been reported to exhibit extreme thermal resistance, and titers of those phages are not affected significantly after (short time) pasteurization (Atamer et al., 2009, 2011; Capra et al., 2013). The distinctly divergent inactivation lines of a heat-sensitive *L. lactis* phage (phage P008) and of a heat-resistant phage (phage P680) are shown in **Figure 1A** for different suspension media (milk, whey, and whey products). The relevant temperature/time areas for microparticulation processes, heat treatment of whey cream and high temperature/short time (HTST) pasteurization of milk are also indicated in **Figure 1A** for orientation.

The 99% reduction of phages in milk by pasteurization at 72°C requires long thermal treatment times between 2 and 300 min (Quiberoni et al., 1999, 2003; Binetti and Reinheimer, 2000; Suárez and Reinheimer, 2002; Capra et al., 2004; Müller-Merbach et al., 2005; Atamer et al., 2009, 2011; Ebrecht et al., 2010; Marvig et al., 2011). For phages of different lactic acid bacteria, the experimental data for this 2-log reduction at 72°C are summarized in **Table 1**.

**Table 1:** Thermal resistance of bacteriophages infecting lactic acid bacteria.

Phage	Heating medium	Host species	*t*_99_ at 72°C (= 2·;D_72°C_) (min)	Source
P680	SM	*Lactococcus lactis*	300	Atamer et al. (2009)
P793	SM	*Leuconostoc pseudomesenteroides*	259	Atamer et al. (2011)
CNRZ 832-B1	RSM	*Lactobacillus helveticus*	21	Quiberoni et al. (1999)
001	RSM	*Lactococcus lactis*	20	Suárez and Reinheimer (2002)
P635	SM	*Lactococcus lactis*	17	Marvig et al. (2011)
0BJ	RSM	*Streptococcus thermophilus*	12	Binetti and Reinheimer (2000)
P008	SM	*Lactococcus lactis*	8.4	Müller-Merbach et al. (2005)
lb_3_	RSM	*Lactobacillus delbrueckii*	2.9	Quiberoni et al. (2003)
Cb1/204^a^	RSM	*Lactobacillus delbrueckii*	2.4	Ebrecht et al. (2010)
J-1	RSM	*Lactobacillus casei*	~2	Capra et al. (2004)
PL-1	RSM	*Lactobacillus paracasei*	~2	Capra et al. (2004)

*For each study, the phage showing the highest heat resistance is given.*

a A temperate *Lb. delbrueckii* phage.

*SM, skim milk; RSM, reconstituted non-fat skim milk; *t*_99_, time to achieve 99% inactivation (2-log reduction; *t*_99_ = 2·;D_72°C_).*

Lactococcal phages were detectable after milk was pasteurized and then spray dried. No reduction in the phage titer was observed during 9-month storage of milk powder (Chopin, 1980) demonstrating the high stability of the phage populations in the dry powder matrix. Evidence for protective effects of milk (components) on phage populations have been published previously (Daoust et al., 1965; de Fabrizio et al., 1999; Suárez and Reinheimer, 2002; Müller-Merbach et al., 2005). In our study on *Leuconostoc* phages, a 1-min heat treatment at 70°C on the test phage P808 resulted in a low phage number reduction (e.g., 1 log unit) when phage was suspended in milk but in a high phage titer decrease (e.g., 4 log units) when phage was suspended in water (Atamer et al., 2011). From comparative thermal inactivation trials conducted with milk and whey it was concluded that the inactivation efficiency of lactococcal phages in whey, whey protein concentrate and whey cream was similar to phage destruction in milk (Atamer and Hinrichs, 2010). It is obvious from the data presented in **Figure 1** that heating conditions usually applied to milk fortified with whey powder for yogurt manufacture (i.e., 90–100°C for up to 5 min; Kessler, 2006) or for production of fresh cheese (i.e., 85–95°C for 3–5 min; Kessler, 2006) cannot warrant complete inactivation of thermo-resistant phages originated from whey powder ingredients. This scenario is particularly problematic for both – mesophilic and thermophilic – starter cultures, since these cultures are frequently attacked by thermo-resistant phages (Atamer et al., 2009, 2011; Capra et al., 2013).

## RECYCLING OF WHEY

For industrial production of various cheese types such as mozzarella, semi-hard and hard cheeses, both – mesophilic and thermophilic – starter cultures are used. Depending on the type of cheese, approximately 3–13 L of whey kg^-1^ of produced cheese is drained (**Table 2**). Whey drained from fresh cheese during production has a pH less than 4.6 (“sour whey”) and a lower content of whey proteins than “sweet whey” obtained from the manufacture of ripened cheeses (pH generally above 6.3; Walstra et al., 2006). Due to its higher amounts of whey proteins, sweet whey batches can be processed into different whey products (Hinrichs, 2001).

**Table 2:** Amount of whey drained from different type of cheese productions.

	Hard cheese	Semi-hard cheese	Soft cheese	Sour milk cheese	Fresh cheese
Liter milk for 1 kg cheese	13–14	12–13	8–9	5–6	4–5
Drained whey (L)	12–13	11–12	7–8	4–5	3–4
Drained whey (%)	92–93	91–92	86–89	80–83	75–80

**FIGURE 2:**
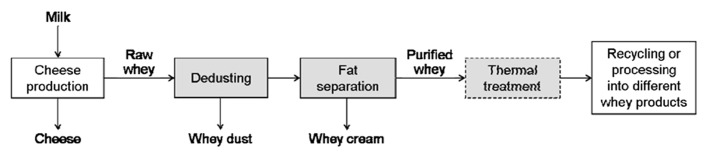
** Process steps of whey in cheese production before its recycling or further processing**.

The main process steps for the refining of cheese whey (before its recycling to the cheese milk or its processing into different products) are illustrated in **Figure 2**. At first, cheese dust (containing small cheese particles) has to be removed from raw whey collected from the production by filters and decanters. In the following step, cream is separated from whey (whey separators). Finally, the whey is heat-treated in order to inactivate the indigenous residual population of lactic acid bacteria (preventing subsequent acidification during storage or further processing of the whey).

**FIGURE 3:**
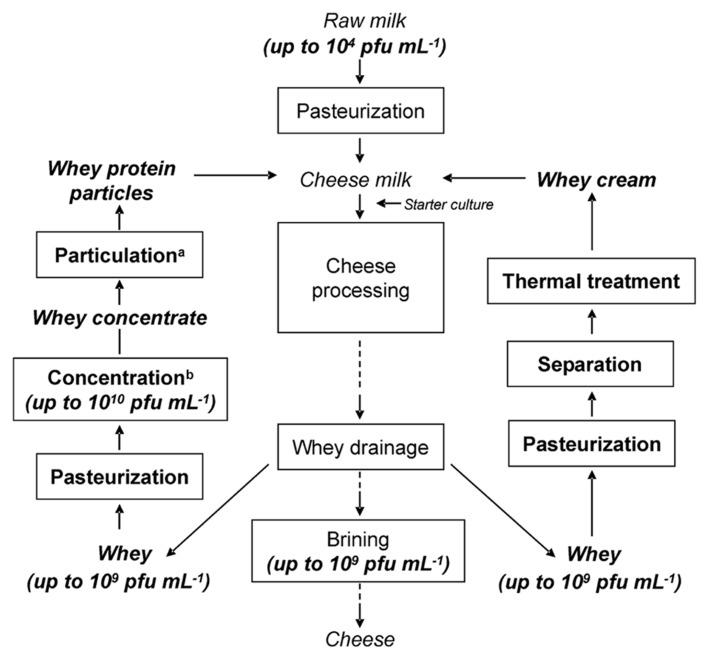
**. Flow chart of a cheese-making process in which concentrated whey proteins and whey cream are being recycled**. ^a^A combination of heating and shearing is applied to whey protein concentrate, ^b^whey is concentrated by means of ultrafiltration (Atamer et al., 2012).

Drained cheese whey is commonly contaminated with phages (Bruttin et al., 1997; Madera et al., 2004; Atamer et al., 2009, 2011). Phages persist in raw milk, in the factory environment and may also originate from starter cultures (Marco et al., 2012). We have shown that thermo-resistant phages are wide-spread and belong to natural phage populations in dairies (Atamer et al., 2009). Raw milk, as the main source of phages in dairies, can be contaminated with up to 10^4^ phages mL^-1^ (McIntyre et al., 1991). The amount of phages in whey can reach numbers as high as 10^9^ pfu mL^-1^ (Atamer et al., 2009, 2011). After concentration of whey, this number will increase by a factor of 10, resulting in phage titers of up to 10^10^ pfu mL^-1^ in the concentrate (if 10% concentration and a maximum phage titer of 10^9^ pfu mL^-1^ are taken into account), making the elimination of phages more difficult. In cheese processing, whey proteins and whey fat can be recycled to increase cheese yield (Kosikowski, 1979; Brown and Ernstrom, 1982; Matthews, 1984; Punidadas et al., 1999; Hinrichs, 2001) or to enhance texture properties (Lawrence et al., 1987). Possible ways for recycling of whey are demonstrated in **Figure 3**, in which a thermal treatment of whey is needed before recycling. After optional thermal treatment of the whey for storage until further processing, whey cream is separated from the whey and the whey is thermally treated before adding to new cheese milk batches. For incorporating whey proteins into cheese, fat-free whey is concentrated by ultrafiltration up to or exceeding 10% protein (see **Table 3**; Bylund, 1995), and is furthermore particulated by heating and shearing. The subsequent heat treatment conditions are summarized in the following section.

**Table 3:** Applied heat treatments for whey and whey products.

Source	Raw material	Protein (%)	Lactose (%)	pH	Temperature/time combination
Singer et al. (1988)	WPC	15–25	5–5	3.5–4.5	80–120°C/3–300 s
Fang and Snook (1991)	WPC	15–25	5–15	5.5–6.9	70–120°C/3 s to 20 min
Asher et al. (1992)	WPC	4–5	4–5	6.0–6.5	75–90°C/5–60 s
Queguiner et al. (1992)	WPI	20	0.1	3.5–3.9	90–100°C/50–100 s
Paquin et al. (1993)	WPC	4–5	<0.2	2.5–7.0	80–120°C/4–600 s
Spiegel (1999); Spiegel and Huss (2002)	WPC	5–20	1–20	3.5–6.7	75–130°C/10 s to 150 min
Huss and Spiegel (2000)	Whey	0.5–2	4–5	5.0–7.0	75–150°C/10 s to 150 min

*WPC, whey protein concentrate; WPI, whey protein isolate.
*

## THERMAL TREATMENT OF WHEY AND INACTIVATION OF PHAGES IN WHEY

Before recycling of whey cream and whey protein particles into cheese milk, a heat treatment is commonly applied to whey in order to inactivate the remaining starter bacteria and contaminants (**Figure 3**). In recent years, “microparticulation” processes, in which a combination of heat treatment and high shear treatment is applied to whey protein concentrate, have been installed in dairy factories to recycle the whey proteins present in cheese whey into either cheese milk or other milk products. **Table 3** summarizes the heat treatment conditions for whey and whey products. Microparticulation processes such as ALPMA CreamoProt^TM^, APV LeanCreme^TM^, and Tetra Therm MicroPart^TM^ are available for the utilization and conversion of liquid whey. With the help of these processes, products like Simplesse^®^ and Dairy-Lo^TM^, which are protein-based fat replacers, can be produced. Whey cream (fat content of 25–30%) can be re-used in cheese-making to standardize the cheese milk (Bylund, 1995, 2003) and a heat treatment at 93°C for at least 30 min is suggested before standardization (Scott et al., 1998). The thermal inactivation curves performed in whey are therefore beneficial for the application of such microparticulation processes and the evaluation of process safety. Inadequate heating of whey and whey derivatives, which will then be recycled into cheese manufacturing process, may cause the emergence of concentrated phage population in dairy environments as well as in milk products. To eliminate the risk of fermentation failure after recycling of whey, a heat treatment ensuring 9-log reduction of phages should be applied, in particular in the presence of heat-resistant phage populations. For the thermal treatment of whey and whey products, 9-log reduction lines of thermo-resistant phages were calculated and proposed when designing a heat treatment process (Atamer and Hinrichs, 2010). The inactivation kinetics of two lactococcal phages (heat-sensitive phage P008 *versus* heat-stable phage P680) were studied in whey in detail. According to the obtained kinetic data, a 9-log reduction of the reference phage P008 was obtained with temperature and time combinations ranging from 70°C for 20 min to 90°C for 1 s. Contrarily, the temperature and time combinations for the same reduction of the thermo-resistant phage P680 were much harsher and ranged from 100°C for 20 min to 140°C for 2 s. Since thermal treatments with such severe temperature/time combinations are not applicable due to the effect they might cause on the functional properties of the product, non-thermal strategies have to be considered for phage elimination.

## UV TREATMENT IN WHEY

UV-C irradiation for inactivation of microorganisms is a powerful methodology for disinfection of surfaces, drinking water and waste water, and has been suggested as an alternative for heat treatment processes (Chang et al., 1985; Sommer et al., 2001; Hazem, 2002; de Roda Husman et al., 2004; Wang et al., 2004; Schmidt and Kauling, 2007). Sommer et al. (2001) studied the inactivation of bacteriophages in water by means of non-ionizing UV radiation (UV-253.7 nm), and a UV light dose of 750 Jm^-2^ was sufficient for a 4-log inactivation of the most heat-resistant phage in tap water. A coiled UV irradiation tube reactor has been designed for inactivation of viruses and bacteria in cloudy liquid media (Schmidt and Kauling, 2007). Since UV rays cannot penetrate cloudy solutions, UV irradiation has therefore some limitations, although it is a powerful tool in inactivating phages. In the literature, a new technology, the UVivatec^®^ process has been described, making UV technology applicable in the cases of very cloudy solutions. In this UVivatec system, a UV lamp is centrally fixed in a tube, resulting in a new concept of flow guidance, in which the flow passes the UV lamp helically (instead of linearly) through a coiled channel. As an alternative to commonly applied heat treatments, UV technology may be considered for potential applications in bacteriophage inactivation in recycling of whey in the future, provided a legal basis for application will be established.

## MEMBRANE FILTRATION OF PHAGES IN WHEY

Membrane separation has been already used in milk processing for several decades, and nowadays microfiltration systems are widely implemented in the dairy industry. In order to separate suspended particles and microorganisms in milk, membranes with pore sizes of approximately 1 μm are used (Bylund, 1995, 2003; Heino, 2009; Schmidt et al., 2012), but for the fractionation of milk proteins into casein and whey protein fractions, membrane pore sizes ranging from 0.05 to 0.2 μm are required. In a recent study, Piry et al. (2012) have focused on improvements of the microbiological quality of whey protein concentrates based on microfiltration procedures. In this study, it was shown that separation of inorganic materials and microorganisms from whey results in the formation of a layer on the membrane (originating from protein aggregates, vegetative microorganisms and spores in whey). These findings stimulated us to examine the separation of dairy phages from whey by membrane filtration technology with the aim of avoiding significant denaturation of whey proteins. A further reason for examination of filtration technology was that irreversible losses of protein activity are not only induced by rigid thermal treatments but are also expected when alternative technologies like, e.g., high pressure treatments or UV light irradiation are applied.

**FIGURE 4:**
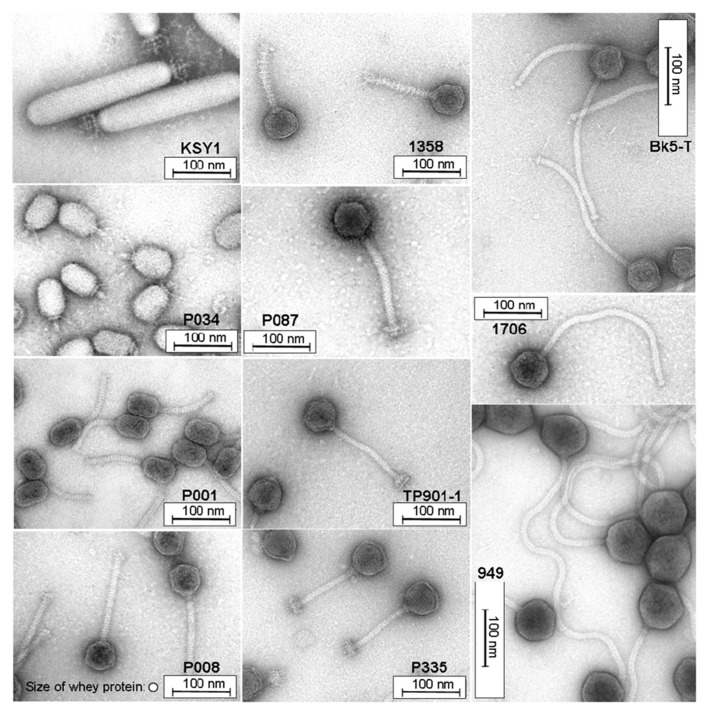
** Transmission electron micrographs of *Lactococcus lactis* phages representing different morphotypes and lactococcal phage species**. For comparison, the size of whey protein is also shown at the bottom of the phage P008 micrograph.

**FIGURE 5:**
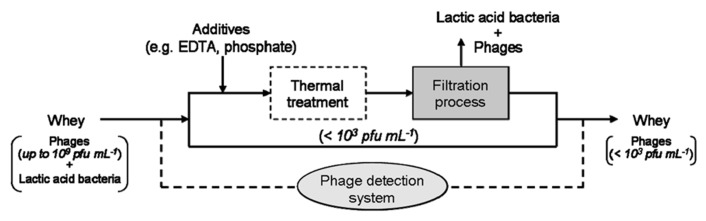
** Example for an application of combined treatments for phage elimination in whey**.

Ultrafiltration is usually applied to concentrate the whey proteins, and cut-off values ranging between 20 and 40 kDa are employed (Heino, 2009). In order to asses the filtration efficiency for lactococcal phages, the different morphotypes and sizes of different phage species have to be considered. Representative phages are shown in **Figure 4** with isometric- and prolate-shaped heads of different dimensions (diameters ranging from approximately 50 to 75 nm). Similar variation of size and morphotype is also illustrated for the (non-contractile) phage tails and baseplate structures (tail lengths varying from 120 to 450 nm). Phage particle dimensions are larger than the protein sizes of the major whey protein fractions with β-lactoglobulin and α-lactalbumin (size: 3–6 nm), but they are comparable with the dimensions of the casein micelles in milk (approximately 50–300 nm; Walstra et al., 2006). Therefore, it must be considered that not only milk proteins (casein as well as whey protein fractions with β-lactoglobulin and α-lactalbumin) but also phage particles are retained by ultrafiltration membranes with a cut-off value of 20 kDa (Mistry and Kosikowski, 1986). Hence, when this retentate is recycled (**Figure 5**), phages would also be added to cheese milk.

In a recent study, it was shown that by using a larger pore size of 300 kDa it is possible to have a permeation of major whey proteins α-lactalbumin and β-lactoglobulin (which are significantly smaller than the minor whey proteins serum albumin, immunoglobulin G, and lactoferrin) and a retention of phages (Almécija et al., 2007). Application of such membranes with larger pore sizes raises the question whether separation of phages in whey is possible while preserving the major whey proteins in whey permeate. In this way one could use the permeate, which contains α-lactalbumin and β-lactoglobulin, instead of the retentate containing phages.

In water treatment facilities, it has already been shown, that a complete retention of poliovirus particles (28–30 nm in diameter, initial value 10^4^/mL) from water can be achieved by ultrafiltration with membranes (cut-off: 30 kDa, polysulfone membranes; Madaeni et al., 1995). With microfiltration membranes (hydrophobic membranes) having 0.2 μm pore sizes a complete retention of poliovirus particles was not observed in the same study. Notably, the retention efficiency could be improved by the presence of biomass (of *Escherichia coli* cells) in the feed solution. The authors concluded that the bacterial biomass resulted in a blocking and obstructing of the membrane pores. The unspecific adsorption of virus particles to the bacterial surfaces apparently led to an effective filtration barrier as a secondary layer on the membrane surface.

Studies with bacteriophage λ and corresponding *E. coli* host cells confirmed that bacteria/phage interactions are crucial for removal of virus particles by microfiltration membranes with 0.2 μm pore sizes (Madaeni and Khodadadi, 2004). Hence, a better retention of viruses was obtained, when the feed solution contained both components (i.e., phages and bacterial host cells). For optimal retention, surface charges of phages and filtration membrane should be either opposite in charge or small in magnitude. The retention behavior of lactococcal phages of different morphotypes across a 0.1-μm pore size membrane has been analyzed in skim milk, and high phage retention rates were shown to be independent of the phage titer (Gautier et al., 1999). Only low numbers of the inoculated phages (0.4–0.14%) were still detectable in the microfiltrate of the casein-deficient milk.

## COMBINED EFFECTS OF DIFFERENT TREATMENTS ON PHAGE ELIMINATION IN WHEY PRODUCTS

Thermal treatment is the most commonly applied method by dairy manufacturers to inactivate phages in whey. However, destruction of thermostable dairy phages is neither assured by low temperature/long time (LTLT) nor by high temperature/short time (HTST) pasteurization conditions. Therefore, for practical reasons, it is advisable to combine different phage inactivation methods rather than applying them separately at extreme conditions, i.e., combined application of thermal and non-thermal methods. Using a membrane filtration process, the amount of phages present in whey can be reduced. By the separation of the host cells of lactic acid bacteria, phage multiplication on the filtration membrane can also be prevented. The aim should be to remove bacteria from whey and to reduce the phage titers in whey to a level below 10^3^ pfu mL^-1^, so that the required heating conditions can be decreased in order to avoid a high heat load on the product. In microfiltration processes of milk, membranes with pore sizes of approximately 100 nm to 1 μm are commonly used (Bylund, 1995, 2003). A filtration process with 100 nm pore size retains both the present lactic acid bacteria and some of the phages. Since the main whey proteins (i.e., β-lactoglobulin and α-lactalbumin) are much smaller, they permeate through microfiltration. One currently investigated approach of combining different inactivation methodologies is illustrated in **Figure 5**. This phage inactivation model may also be expanded by additives (e.g., EDTA, phosphate) before filtration or by an auxiliary mild heat treatment (<70°C), which could have an effect on aggregation of phages and therefore on their separation (Atamer et al., 2010). For the problematic (i.e., thermo-resistant) lactococcal phages, sensitive PCR phage detection systems can be used to recognize phage accumulation in due time (Atamer et al., 2009).

## CONCLUSION

Optimization of production processes for cheese and fermented milk and reutilization of whey obtained from the cheese production are important factors for the economic success of dairy companies. Whey can be transformed into various native whey protein supplements or directly used in different dairy products. However, cheese whey usually contains high numbers of phages that have to be eliminated before reutilization of whey. Different treatments are applied to remove phages from the process. Heat treatment is the most commonly applied method, however, non-thermal treatments such as membrane separation and UV treatment are also available as alternatives to thermal treatment. Application of combination of these methods is suggested rather than using them separately at extreme conditions. A membrane filtration process may be used together with a thermal process in the reduction or elimination of thermo-resistant phages in whey, since the application of a thermal process alone has some limitations due to heat sensitivity of whey proteins. Minimizing or even eliminating the fermentation problems caused by the reutilization of whey and whey products is of great importance for the dairy companies in enhancing their productivity and their competitive position. Future efforts should therefore focus on the separation of phages from whey in combination with further phage-reducing methods prior to the filtration process.

## Conflict of Interest Statement

The authors declare that the research was conducted in the absence of any commercial or financial relationships that could be construed as a potential conflict of interest.
